# Echinococcal infection of the liver and the lung

**DOI:** 10.11604/pamj.2024.48.74.43696

**Published:** 2024-06-27

**Authors:** Ashwin Karnan

**Affiliations:** 1 Department of Respiratory Medicine, Datta Meghe Institute of Higher Education and Research, Sawangi (Meghe), Wardha, Maharashtra, India

**Keywords:** Hydatid, cough, tapeworm, echinococcosis

## Image in medicine

A 62-year-old female presented to the outpatient department with complaints of cough, breathlessness, and right-sided chest pain for the past 10 days. The patient gave a characteristic salty taste of sputum and a history of having a pet dog for 3 years. Computed tomography (CT) scan of the thorax and abdomen showed a cavitary lesion in the right lower lobe with communication to the right pleural space, right-sided effusion, and a hypodense lesion in segment VII of the liver with a calcified wall. Serum IgG for Echinococcus granulosus was positive confirming the diagnosis of hepatopulmonary hydatidosis. The patient was treated with a 90-day course of albendazole and is currently on follow-up and planned for total pericystectomy. Hydatid disease is a zoonotic disease caused by species belonging to Echinococcus. The most common organs involved include the liver and the lungs. Surgery is the treatment of choice for both pulmonary and hepatic hydatid cysts but in inoperable cases, medical management may be tried.

**Figure 1:**
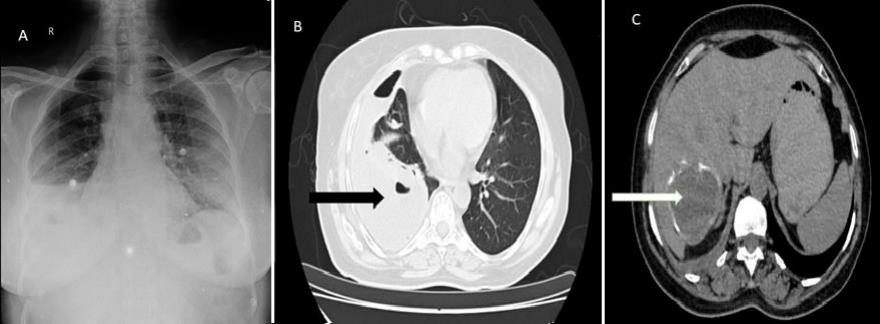
A) chest X-ray of the patient showing right pleural effusion; B) computed tomography (CT) of the thorax with a black arrow showing intrapulmonary hydatid cyst with pleural extension; C) CT of the abdomen with a white arrow showing hepatic hydatid cyst

